# The effects of synergistic blend of organic acid or antibiotic growth promoter on performance and antimicrobial resistance of bacteria in grow–finish pigs

**DOI:** 10.1093/tas/txaa211

**Published:** 2020-11-27

**Authors:** Tran Thi Bich Ngoc, Duong Thi Oanh, Lane Pineda, Suparlark Ayudhya, Nienke de Groot, Yanming Han

**Affiliations:** 1 Department of Animal Feed and Nutrition, National Institute of Animal Science, Ha Noi, Vietnam; 2 Trouw Nutrition R&D, Amersfoort, The Netherlands; 3 Trouw Nutrition, Bangkok, Thailand

**Keywords:** antibiotic growth promoter, antimicrobial resistance, *E. coli*, grow, finish pigs, performance, Selacid GG

## Abstract

This study was carried out to evaluate the effect of Selacid Green Growth (GG) or antibiotic growth promoter (AGP) on the performance and economics of grow–finish (GF) pigs. The Selacid GG is a blend of short-chain fatty acids (formic acid, acetic acid, lactic acid, propionic acid, citric acid, and sorbic acid), buffered organic acid (ammonium formate), and a combination of medium-chain fatty acids (C8, C10, and C12). A total of 312 grower pigs (Yorkshire × Landrace × Duroc) with initial body weight (BW) of 26.5 ± 0.92 kg were used in a 90-d feeding trial. The pigs were allocated randomly to three treatments consisting of eight replicate pens with 13 pigs each. The treatments tested included a 1) negative control (control): basal diet without colistin and Selacid GG, 2) positive control (AGP): basal diet with colistin (20 g/ton), and 3) Feed additive (Selacid GG): basal diet with Selacid GG (2 kg/ton). The results showed that, over the entire period of the experiment, the dietary supplementation of Selacid GG elicited a similar effect as AGP on feed cost and on all growth parameters measured (*P* > 0.05). In relation to the control group, Selacid GG significantly improved the final BW (+3.4 kg or 3.6%), average daily gain (+39 g/pig or 5.3%), and gain:feed (+30 g or 8.1%) of pigs (*P* < 0.05). In addition, the feeding of Selacid GG reduced feed cost (−0.078 USD) per kilogram BW gain. The average daily feed intake was not affected by dietary treatments (*P* > 0.05). *Escherichia coli* was prevalent in 46 out of 48 fecal samples tested. All *E. coli* isolates were resistant to colistin, amox-colistin, ciprofloxacin, and enrofloxacin. The number of *E. coli* isolates resistant to amoxicillin/clavulanic, cefotaxime, ceftiofur, and norfloxacin was significantly reduced, whereas the inhibitory zones of amocxillin/clavulanic acid were increased; and the susceptibility of *E. coli* to amoxcillin/clavulanic, cefotaxime, ceftiofur, ciprofloxacin, nofloxacin, and flumequin was increased when Selacid GG was added in the feeds (*P* < 0.05). The findings of the study suggest that Selacid GG is a cost-effective product with the same efficacy as AGP in promoting the growth and economic performance of GF pigs. The product is safe and can be added to the diet of GF pigs without developing resistance to selected antibiotics.

## INTRODUCTION

More recent studies, conducted after 2000, suggest that productivity gains from antibiotic growth promoters (AGP) in swine production are lower compared with earlier research conducted before 2000 (Laxminarayan et al., 2015). For instance, Miller et al. (2003) reported that the use of AGP increased average daily gain (ADG) by 0.5% and feed efficiency by 1.1% in grow–finish (GF) pigs, which is much lower than the improvements reported in the 1980s (Cromwell, 2002). One of the possible reasons for the reduction in growth response can be the optimization of production conditions in the swine industry or an increasing level of resistance of bacteria to certain types of antibiotics. Due to these tendencies, Sweden first prohibited the use of some of the antibiotics in animal feeds in 1986, and European Union member nations banned all AGP in 2006 according to European Parliament and Council Regulation EC No. 1831/2003. This cascade of events around the use of antibiotics stimulated a search for effective products and ingredients to replace AGP. Alternative molecules that have been used include organic acids, probiotics, prebiotics, enzymes, medium-chain fatty acids, essential oils, yeasts, zinc, and plant extracts. However, among all these alternatives, blends of organic acids have been broadly applied worldwide with reasonable success (Mroz, 2005). Positive effects of acids are associated mainly with increased gastric acidity, antibacterial activity, reduced coliform populations, and improved digestibility (Jensen et al., 2003; Devi et al., 2016), resulting in an improvement in the performance and feed efficiency of fattening pigs (Partanen and Mroz, 1999; Overland et al., 2000; Devi et al., 2016). Several studies show that certain organic acids can inhibit the growth of multiresistant bacteria and that they can act against bacterial biofilms, which cannot be achieved by antibiotics (Goualié et al., 2014; Akbas and Cag, 2016). To study the emergence of antibiotic resistance (AR) in gram-negative bacteria, *Escherichia coli* are widely accepted as indicator bacteria (Kaesbohrer et al., 2012). They are commensal members of the normal gastrointestinal microbiota in humans and animals, can be rapidly altered by exposure to antibiotics, according to Francino (2016), and act as an important pool of resistance determinants (Schjorring and Krogfelt, 2011). Therefore, this study was conducted to validate the efficacy of a blend of short-chain fatty acids (formic acid, acetic acid, lactic acid, propionic acid, citric acid, and sorbic acid), buffered organic acid (ammonium formate), and a combination of medium-chain fatty acids (C8, C10, and C12) on the growth and economic performance of GF pigs. Furthermore, the study investigated the antimicrobial resistance of *E. coli* isolated in feces of GF pigs.

## MATERIALS AND METHODS

The Scientific Committee at the National Institute of Animal Science—Vietnam reviewed and approved the protocol of this research, including animal care and use.

### Animals and Treatments

A total of 312 grower pigs (Yorkshire × Landrace × Duroc) with initial body weight (BW) of 26.5 ± 0.92 kg was used in a 90-d feeding trial. The pigs were blocked according to gender and allocated to three treatments consisting of eight replicate pens (three pens male and five pens female) with 13 pigs each. Initial BW and ancestry were equally distributed across treatments. The experiment was repeated in three batches to meet the desired number of replicates.

The treatments tested included a 1) negative control (control): basal diet without colistin and Selacid Green Growth (GG), 2) positive control (AGP): basal diet with colistin (20 g/ton), 3) feed additive (Selacid GG): basal diet with Selacid GG (2 kg/ton). The Selacid GG is a free-flowing powder based on a blend of synergistic short-chain fatty acids (formic acid, acetic acid, lactic acid, propionic acid, citric acid, and sorbic acid), buffered organic acid (ammonium formate), and a combination of medium-chain fatty acids (C8, C10, and C12) for a broad-spectrum effect in the gut.

### Experimental Diets

An AGP-free diet based on corn, wheat, and soybean was formulated to meet the nutrient requirements of GF pigs according to NRC (1998) and offered in a pelleted form (Table 1). A masterbatch of the basal diet using the same batch of ingredients was prepared in mash form, then the colistin (20 g/ton) and Selacid GG (2 kg/ton) were added on top of the basal diet, mixed well, pelleted, and, thereafter, put into premarked feed bags. After the individual feed was made, a representative sample was taken from each dietary treatment for the determination of moisture, crude protein, Ca, and P. Dry matter (967.03), total N (984.13), Ca, and P were analyzed according to the standard AOAC methods (Association of Official Analytical Chemist [AOAC], 1990).

**Table 1.** Ingredient and nutrient composition of the basal diet

Ingredient, kg	Growing (25–60 kg)	Finishing (60–100 kg)
Corn	449.8	479.8
DDGS corn	115	110
Wheat	100	100
Wheat bran	120	150
Soybean meal	180	120
Soybean oil	15	24
L-lysine	0.5	1
DL-methionine	1.5	1
Salt	5	5
Di-calcium phosphate	15	12
Vitamin–mineral premix^*a*^	2.5	2.5
Phytase	0.2	0.2
Cost of basal diet (USD/kg)	0.401	0.348
Nutrient content of the diets		
Dry matter, %	89.22	89.32
ME^*b*^, Kcal/kg	3203	3205
Crude protein, %	18.08	16.06
Digestible lysine^*b*^, %	0.70	0.59
Digestible methionine + cysteine^*b*^, %	0.49	0.39
Digestible threonine^*b*^, %	0.51	0.38
Digestible tryptophan^*b*^, %	0.16	0.14
Calcium, %	0.53	0.43
Phosphorus avail, %	0.26	0.22

^*a*^Vitamin–mineral premix provided the following quantities of vitamins and minerals per kilogram on air-dry basis: vitamin A, 2,000 IU; vitamin D_3_, 400 IU; vitamin E, 12.0 IU; vitamin K, 1.42 mg; vitamin B_2_, 0.96 mg; vitamin B_12_, 0.03 mg; vitamin B_5_, 3.2 mg; vitamin PP, 4.8 mg; iron, 25.4 mg; zinc, 37.0 mg; manganese, 11.8 mg; copper, 36.0 mg; iodine, 0.15 mg; cobalt, 0.1 mg; Selenium, 0.12 mg.

^*b*^Calculated values.

### Management

Pigs were housed in an open-sided building with concrete flooring. Each pen measures 4 × 5 m in size and is equipped with one feeder and a nipple drinker to provide pig access to feed and water ad libitum. The pelleted feed was put on feeder twice per day, at 800 and 1430 h.

### Measurements and Records

Pigs were monitored for general health and performance. To calculate feed intake, the amount of feed offered and refusal was recorded daily throughout the duration of the experiment. The pigs were weighed at the beginning and at the end of the experimental period before the morning feeding. Gain:feed was calculated as BW gain per kilogram of feed intake. The cost per kilogram BW gain was calculated at the end of the experiment.

#### Antibiogram test.

On the last day of each phase, fresh fecal samples (100 g) were taken from two pigs randomly selected per pen, which were pooled and subsampled (100 g). A total of 48 fecal samples were analyzed for the susceptibility and/or resistance of *E. coli* to different types and groups of antibiotics. The isolates were tested for susceptibility against colistin (10 µg), amox-colistin (10/10 µg), amoxicillin/clavulanic acid (20/10 µg), cefotaxime (30 µg), ceftiofur (30 µg), ciprofloxacin (5 µg), enrofloxacin (5 µg), flo-doxy (40/20 µg), flumequin (30 µg), norfloxacin (10 µg), and pen-strep (15/15 µg) on Mueller–Hinton agar plates by disc diffusion method. The inhibitory zones around the antibiotic discs were measured with a ruler in millimeters. The sensitivity and resistance of the isolates toward the antibiotics were determined as per the criteria of the Clinical and Laboratory Standards Institute (CLSI, 2014) and according to actual Vietnam conditions: resistant (R), inhibitory zones ≤12 mm; low susceptible, 13 mm ≤ inhibitory zones ≤ 12 mm; intermediate susceptible, 17 mm ≤ inhibitory zones ≤ 20 mm; high susceptible, inhibitory zones ≥21 mm.

### Statistical Analysis

The data on performance were analyzed using PROC MIXED in SAS considering the fixed effect of treatment and batch as a random effect. Gender was included in the model as a covariate to correct for the effect of gender. Differences among treatments were separated by Tukey test. The significance level was considered at *P* < 0.05. The antibiogram data were analyzed using chi-square.

## RESULTS

### Growth Performance

During the growing period, pigs fed diets with Selacid GG and AGP showed an increased ADG and gain:feed compared to pigs given control diets (*P* < 0.05; Table 2), while the average daily feed intake (ADFI) was not affected by the treatments during this period of growth (*P* > 0.05; Table 2). However, in the finishing period, the dietary treatments did not influence any of the growth parameters measured (*P* > 0.05; Table 2).

**Table 2.** Effect of treatment on pig performance during the growing and finishing periods and overall

Parameter	Control	AGP	Selacid GG	SEM	*P*-value
Growing period (25–60 kg)					
ADG, g	667.87^b^	713.08	728.68	19.739	0.0003
ADFI, kg	1.56	1.53	1.54	0.061	0.830
Gain:feed	431.65^a^	469.43^b^	476.76^b^	17.875	0.010
Finishing period (60–100 kg)					
ADG, g	837.92	845.13	850.86	27.091	0.910
ADFI, kg	2.65	2.62	2.57	0.067	0.390
Gain:feed	315.32	321.18	326.87	19.031	0.790
Overall period (25–100 kg)					
Start weight, kg	26.79	26.74	26.675	1.630	0.700
End weight, kg	92.49^a^	94.94^b^	95.84^b^	1.726	0.0004
ADG, g	741.17^a^	769.35^b^	780.20^b^	18.729	0.0001
ADFI, kg	2.03	2.00	1.98	0.062	0.030
Gain:feed	366.07^a^	385.70^b^	395.71^b^	12.042	0.001
Mortality rate^*a*^, %	8.7	6.7	5.8	—	0.068

^*a*^Pig mortality was analyzed with chi-square. The reason of death was pneumonia.

^a,b^Values in a row with no common superscripts differ significantly (*P* ≤ 0.05).

Overall, the dietary supplementation of Selacid GG elicited a similar effect as AGP on all growth parameters measured (*P* > 0.05; Table 2). In relation to the control group, Selacid GG significantly improved the final BW (+3.4 kg or 3.6%), ADG (+39 g/pig or 5.3%), and gain:feed (+30 g or 8.1%) of pigs (*P* < 0.05). The ADFI was not affected by the dietary treatments (*P* > 0.05; Table 2). During experimental time, pig fed Selacid GG had a trend to reduce mortality rate; however, it was not different among diets (*P =* 0.068).

### Cost Per Kilogram BW Gain

The feed cost to produce kilogram BW gain was lower in Selacid GG (0.957 USD) and AGP (0.991 USD) groups compared to the control (1.035 USD; Table 3). Feed cost was reduced by 0.078 USD when Selacid GG was used instead of control. The current result demonstrated the economic feasibility of using Selacid GG as a possible replacement for AGP in the diets of GF pigs.

**Table 3.** Feed cost per kilogram BW gain

Parameter	Control	AGP	Selacid GG
Basal diet cost, USD/kg			
At the growing period	0.4010	0.4010	0.4010
At the finishing period	0.3480	0.3480	0.3480
Product cost, USD/kg basal diet			
Selacid GG			0.0029
Colistin		0.0068	
Feed cost, USD/kg	0.3716	0.3785	0.3747
Average feed intake/pig, kg	183.03	178.62	176.55
Total feed cost/pig, USD	68.00	67.61	66.16
Average BW gain/pig	65.70	68.20	69.16
Cost to produce kg BW gain, USD	1.035	0.991	0.957

### Antibiogram Test

A total of 48 samples were collected to analyze the prevalence of *E. coli* and *Salmonella* in the feces of GF pigs. Out of the 48 fecal samples, 46 (95.8%) are positive for *E. coli* and 5 (10.4%) with *Salmonella*.

*E. coli* was isolated in all fecal samples of pigs fed control (8/8) and AGP (8/8) diets, while seven out of eight samples from the Selacid GG-supplemented pigs. *Salmonella* was isolated in the fecal samples of pigs fed control diets during the growing (2/8) and finishing period (3/8). In the Selacid GG-fed pigs, *Salmonella* was detected during the finishing period (2/8) but not during the growing period. No *Salmonella* was isolated in the feces of pigs fed AGP (0/8) in both phases of growth (Table 4).

**Table 4.** Effect of treatment on *E. coli* and *Salmonella* strains isolated from pig feces

	*E. coli*			*Salmonella*		
Parameter	Ʃ	*n*	%	Ʃ	*n*	%
Growing period (25–60 kg)						
Control	8	8	100	8	2	25.0
AGP	8	8	100	8	0	0
Selacid GG	8	7	87.5	8	0	0
Finishing period (60–100 kg)						
Control	8	8	100	8	3	3.75
AGP	8	8	100	8	0	0
Selacid GG	8	7	87.5	8	2	2.5

### Inhibitory Zones

The inhibitory zones of amoxicillin/clavulanic acid and cefotaxime were significantly higher in the *E. coli* isolates of grower pigs fed Selacid GG compared to AGP (*P* < 0.05; Table 5), whereas no significant difference was observed among treatments on the inhibitory zones of other antibiotics (*P >* 0.05). The treatments did not influence the inhibitory zones of tested antibiotics for *E. coli* during the finishing phase of growth (*P* > 0.05; Table 6).

**Table 5.** Effect of treatment on inhibitory zones of *E. coli* isolates in feces growing pigs, mm

Treatment	Control	AGP	Selacid GG	SEM	*P*-value
Colistin (10 µg)	6.25	5.75	5.86	1.401	0.966
Amox-colistin (10/10 µg)	2.38	2.13	3.43	1.711	0.862
Amoxicillin/clavulanic acid (20/10 µg)	15.50^ab^	12.13^b^	17.29^a^	0.936	0.004
Pen-strep (15/15 µg)	7.38	7.88	8.29	1.772	0.940
Flo-doxy (40/20 µg)	11.13	11.75	11.57	1.854	0.970
Flumequin (30 µg)	9.50	12.50	10.86	2.647	0.728
Cefotaxime (30 µg)	19.50^a^	14.38^b^	19.86^a^	1.618	0.048
Ceftiofur (30 µg)	15.50	11.25	16.14	1.895	0.175
Ciprofloxacin (5 µg)	16.63	12.63	16.43	2.064	0.330
Norfloxacin (10 µg)	14.25	14.50	15.29	2.320	0.952
Enrofloxacin (5 µg)	12.13	14.50	14.43	2.550	0.762

^a,b^Values in a row with no common superscripts differ significantly (*P* ≤ 0.05).

**Table 6.** Effect of treatment on inhibitory zones of *E. coli* isolates in feces finishing pigs, mm

Treatment	Control	AGP	Selacid GG	SEM	*P*-value
Colistin (10 µg)	6.12	4.75	6.71	1.327	0.585
Amox-colistin (10/10 µg)	3.38	3.75	4.43	1.825	0.924
Amoxicillin/clavulanic acid (20/10 µg)	13.13	12.00	15.43	1.291	0.210
Pen-strep (15/15 µg)	1.63	3.13	2.71	1.796	0.832
Flo-doxy (40/20 µg)	7.63	6.88	8.43	2.596	0.920
Flumequin (30 µg)	4.38	8.63	7.57	3.016	0.594
Cefotaxime (30 µg)	15.63	16.13	17.29	1.275	0.669
Ceftiofur (30 µg)	12.88	12.38	13.43	1.038	0.789
Ciprofloxacin (5 µg)	9.38	9.50	10.43	2.876	0.964
Norfloxacin (10 µg)	8.25	6.88	8.71	3.006	0.908
Enrofloxacin (5 µg)	8.13	7.38	8.57	3.561	0.973

^a,b^Values in a row with no common superscripts differ significantly (*P* ≤ 0.05).

### Antibiotic Resistant

No significant difference was observed among the treatments on the resistance of *E. coli* to colistin, amox-colistin, pen-strep, ciprofloxacin, and enrolfloxacin in both phases of growth (*P* > 0.05; Table 7). The supplementation of Selacid GG significantly reduced the rates of *E. coli* resistant to amoxicillin/clavulanic, cefotaxime. and ceftiofur during the growing and finishing period compared to the AGP group (*P* < 0.05). In addition, Selacid GG reduced the resistance of *E. coli* to nofloxacin during the finishing period (*P* < 0.05). The feeding of AGP diets, on the other hand, reduced the resistance of *E. coli* to flo-doxy in the growing period but not during the finishing period.

**Table 7.** Antibiotic resistant and susceptibility of *E. coli* isolated in feces of growing–finishing pigs

		Resistant		Highly susceptible	
Antibiotic	Treatment	Growing	Finishing	Growing	Finishing
Colistin (10 µg)	Control	100	100	0	0
	AGP	87.5	100	0	
	Selacid GG	100	100	0	0
	*P*-value	0.581			
Amox-colistin (10/10 µg)	Control	100	100	0	0
	AGP	100	100	0	0
	Selacid GG	85.7	100	0	0
	*P*-value	0.489			
Amoxicillin/clavulanic acid (20/10 µg)	Control	12.5^b^	37.5^a^	12.5^a^	0
	AGP	37.5^a^	50^a^	0^b^	0
	Selacid GG	0^c^	0^b^	14.3^a^	0
	*P*-value	<0.0001	<0.0001	0.001	
Pen-strep (15/15 µg)	Control	75	87.5	0	0
	AGP	87.5	87.5	0	0
	Selacid GG	85.7	100	0	0
	*P*-value	0.576	0.567		
Flo-doxy (40/20 µg)	Control	62.5^a^	62.5^ab^	0	0
	AGP	25^b^	75.0^a^	0	0
	Selacid	42.9^a^	42.9^b^	0	0
	*P*-value	<0.0001	0.013		
Flumequin (30 µg)	Control	50	75	0	0^b^
	AGP	37.5	62.5	0	0^b^
	Selacid GG	42.9	57.1	0	14.3^a^
	*P*-value	0.405	0.273		<0.0001
Cefotaxime (30 µg)	Control	0^b^	25^a^	37.5^c^	12.5^b^
	AGP	25^a^	25^a^	12.5^b^	0^c^
	Selacid GG	0^b^	0^b^	57.1^a^	28.6^a^
	*P*-value	<0.0001	<0.0001	<0.0001	<0.0001
Ceftiofur (30 µg)	Control	12.5^b^	50^a^	0^b^	0
	AGP	50^a^	62.5^a^	12.5^a^	0
	Selacid	14.3^b^	28.6^b^	14.3^a^	0
	*P*-value	<0.0001	0.002	0.001	
Ciprofloxacin (5 µg)	Control	25	62.5	12.5^a^	12.5^a^
	AGP	25	50	0^b^	12.5^a^
	Selacid GG	14.3	57.1	14.3^a^	0^b^
	*P*-value	0.169	0.499	0.001	0.002
Norfloxacin (10 µg)	Control	12.5	62.5^ab^	0^b^	0^b^
	AGP	12.5	75.0^a^	0^b^	12.5^a^
	Selacid GG	14.3	42.9^b^	14.3^a^	0^b^
	*P*-value	0.921	0.013	<0.0001	<0.0001
Enrofloxacin (5 µg)	Control	37.5	50	12.5^a^	12.5
	AGP	25	62.5	12.5^a^	12.5
	Selacid GG	28.6	57.1	0^b^	14.3
	*P*-value	0.256	0.499	0.002	0.921

More data on Low Susceptible and Intermediate Susceptible of E. coli isolated in feces of growing and finishing pigs are presented in supplementary Appendix Table 1 and 2.

^a,b^Values in a column with no common superscripts differ significantly (*P* ≤ 0.05).

Selacid GG increased the susceptibility of *E. coli* to amoxcillin/clavulanic, cefotaxime, ceftiofur, ciprofloxacin, and nofloxacin during the growing period (*P* < 0.05). In the finishing period, the pigs in this group were highly susceptible to flumequin and cefotaxime (*P* < 0.05; Table 7).

## DISCUSSION

Organic acids were reported to stimulate the digestive system of pigs and to improve pig performance because of their antimicrobial activity. The current work showed that Selacid GG was effective as antibiotics in promoting the growth and feed efficiency of pigs. The results suggest that Selacid GG can be used as an alternative to AGP to achieve significant growth performance improvement in GF pigs. The improvement in ADG and gain:feed was possibly achieved due to the antimicrobial activity of organic acid, which helps in the reduction of pathogenic microbial population growth, thereby reducing the metabolic need of microbes and increasing the availability of dietary energy and nutrients to host animals (Upadhaya et al., 2014). Dietary organic acids have been shown to improve the apparent nutrient digestibility in growing pigs (Sauer et al., 2009; Bühler et al., 2010; Machinsky et al., 2015; Xu et al., 2018) and weanling pigs (Guggenbuhl et al., 2007; Halas et al., 2010; Xu et al., 2018). Our findings support previous works from various researchers who reported the positive effect of organic acids on ADG and feed conversion ratio in GF pigs (Falkowski and Aherne, 1984; Giesting and Easter, 1985; Blank et al., 1999; Metzler and Mosenthin, 2007; Suryanarayana et al., 2012). Supplementation of Selacid GG in the pigs had impacts on ADG and gain:feed in the growing period, while ADG and gain:feed were not affected by Selacid GG in the finishing period. This indicated that growth response to Selacid GG was shown to be greater in younger pigs than in older animals. Similarly, in a meta-analysis study conducted by Tung and Pettigrew (2006), the improvements of growth rates were 12.25% and 6.03% for the first 2 and 4 wk postweaning, respectively, while the enhancement was lower for growing (3.51%) or finishing (2.69%) pigs.

The present result indicated that the supplementation of Selacid GG in the diet of GF pigs brought higher economic efficiency than the control group. This can be explained as pigs fed Selacid GG diet had higher BW gain (kg/pig) and lower mortality rate compared to pigs fed control diet during growing–finishing periods (Table 2), thereby feed cost per kilogram BW gain was lower for Selacid GG diet than for control diet.

Our results showed that pigs fed Selacid GG diet gave lower resistance and higher susceptibility frequency to amoxicillin/clavunanic acid, ciprofloxacin in the grower pigs and to amoxicillin/clavunanic acid, cefotaxime, ceftiofur, enrofloxacin, flo-doxy, flumequin, and norfloxacin in the finisher pigs compared to pigs fed control and colistin diets. The ability of organic acids to reduce *E. coli* counts and increase *Lactobacillus* counts in pigs is known (Zentek et al., 2013; Ahmed et al., 2014; Upadhaya et al., 2014; Devi et al., 2016) and, therefore, the reduction of antibiotic-resistant *E. coli* is possible. Intestinal commensal bacteria in both animals and humans are considered good indicators of the general level of antimicrobial resistance as they are exposed to selection pressure driven by any antimicrobial treatment of their host (Blake et al., 2003; Szmolka and Nagy, 2013). According to Roth (2015), the lower the counts of resistant bacteria in the intestinal flora, the lower the possibility that genes encoding resistance will be transferred to other bacteria, including pathogenic bacteria. A study by Roth (2015) showed no difference in the total *E. coli* count between control and feed additive (consisting of organic acids, cinnamaldehyde, and a permeabilizer) pig groups but lower counts of resistant *E. coli* on day 14 of experimental period for feed additive pig group. Besides, at day 42 of the trial, total *E. coli* counts in the fecal samples and the count of *E. coli* resistant to ampicillin in the pig group fed feed additive was, respectively, about 90% and 60% lower than the control group, while the count of *E. coli* with multiresistance to tetracycline, streptomycine, and sulfamethoxazolin in the trial group was nearly 90% below the control group. Similarly, Roth et al. (2017) reported that broiler diet-supplemented organic acids (formic acid, acetic acid, and propionic acid) contributed to a significant decrease in *E. coli* resistant to ampicillin and tetracycline compared to the control and enrofloxacin groups, as well as to a decrease in sulfamethoxazole and ciprofloxacin-resistant *E. coli* compared to the enrofloxacin group. Treatment with enrofloxacin increased the number of *E. coli* resistant to ciprofloxacin, streptomycin, sulfamethoxazole, and tetracycline and extended spectrum beta-lactamase-producing *E. coli* in the ceca of broilers. The results of the studies by Roth (2015) and Roth et al. (2017) supported our above findings. According to Goualié et al. (2014), using organic acids in the poultry production chain can reduce the propagation of antibiotic multiresistant strains of *Campylobacter*. However, an obvious reduction in the quantity of selected antibiotic resistance genes by the acid-based feed additive for weaned piglets was not detected in the study by Wegl et al. (2017), while clear effects of the oxytetracycline feed supplementation on the resistance gene prevalence and quantity were observed. The reduction of antibiotic-resistant *E. coli* or the increase in susceptibility by using Selacid GG suggested that Selacid GG has the potential to treat *E. coli* infection without causing any resistance.

The causes of antimicrobial resistance are complex, but there is growing scientific evidence suggesting that low-dose, prolonged courses of antibiotic use for animal husbandry accelerated the emergence and spread of resistant bacteria (Marshall and Levy, 2011; Mole, 2013; Pruden et al., 2013). This supports the current finding that pigs fed AGP had higher *E. coli* resistance and lower susceptibility to some antibiotics in growing and finishing periods, even pigs fed Colistin at a lower dose of 20 grams/ton. In food animal husbandry, antimicrobial resistance can spread not only by direct contact but also indirectly. Direct effects are those that can be causally linked to contact with antibiotic-resistant bacteria from swine. Indirect effects are those that result from contact with resistant organisms that have been spread through food, water, and animal waste application to soil (Landers et al., 2012).

## CONCLUSIONS

The results of the current study indicated that Selacid GG is a cost-effective product with the same efficacy as AGP in promoting the growth and economic performance of GF pigs. The *E. coli* isolated in the study were multidrug resistant. However, the use of Selacid GG positively influenced the number of resistance and susceptible *E. coli* to selected antibiotics. *E. coli* resistance to amoxicillin/clavulanic, cefotaxime, ceftiofur, and norfloxacin was reduced, whereas the susceptibility of *E. coli* to amoxcillin/clavulanic, cefotaxime, ceftiofur, ciprofloxacin, nofloxacin, and flumequin were increased when Selacid GG was added in the feeds.

The study concluded that the dietary supplementation of Selacid GG could be used as a replacement for AGP to achieve a significant improvement in the growth and economic performance of fattening pigs. Selacid GG is a safe product that can be added in the diets of GF pigs without developing resistance to selected antibiotics.

## Supplementary Material

txaa211_suppl_Supplementary_Materials
