# Supplementary material for: The effects of synergistic blend of organic acid or antibiotic growth promoter on performance and antimicrobial resistance of bacteria in grow–finish pigs
Source: Transl Anim Sci. 2020 Nov 27;4(4):txaa211. doi: 10.1093/tas/txaa211 (PMC7770621; doi:10.1093/tas/txaa211)
Supplement: txaa211_suppl_Supplementary_Materials [file txaa211_suppl_supplementary_materials.docx]

**Appendix Table 1.** Antibiotic resistant of *E. coli* isolated in feces of growing pigs, %

| Antibiotics | Treatment | R | LS | IS | HS |  | Antibiotics | Treatment | R | LS | IS | HS |
| --- | --- | --- | --- | --- | --- | --- | --- | --- | --- | --- | --- | --- |
| Colsitin (10 µg) | Control | 100 | 0^b^ | 0 | 0 |  | Cefotaxime (30µg) | Control | 0^b^ | 12.5^b^ | 50^a^ | 37.5^b^ |
|  | AGP | 87.5 | 12.5^a^ | 0 | 0 |  |  | AGP | 25^a^ | 37.5^a^ | 25^b^ | 12.5^c^ |
|  | Selacid GG | 100 | 0^b^ | 0 | 0 |  |  | Selacid GG | 0^b^ | 14.3^b^ | 28.6^b^ | 57.1^a^ |
|  | *P-value* | 0.581 | <0.0001 |  |  |  |  | *P-value* | <0.0001 | <0.0001 | 0.005 | <0.0001 |
| Amox –Colistin (10/10µg) | Control | 100 | 0^b^ | 0 | 0 |  | Ceftiofur (30µg) | Control | 12.5^b^ | 50 | 37.5^a^ | 0^b^ |
|  | AGP | 100 | 0^b^ | 0 | 0 |  |  | AGP | 50^a^ | 37.5 | 0^c^ | 12.5^a^ |
|  | Selacid GG | 85.7 | 14.3^a^ | 0 | 0 |  |  | Selacid GG | 14.3^b^ | 42.9 | 28.6^b^ | 14.3^a^ |
|  | *P-value* | 0.489 | <0.0001 |  |  |  |  | *P-value* | <0.0001 | 0.405 | <0.0001 | 0.001 |
| Amoxicillin/clavulanic acid (20/10µg) | Control | 12.5^b^ | 50 | 25^a^ | 12.5^a^ |  | Ciprofloxacin (5µg) | Control | 25 | 12.5^b^ | 50 | 12.5^a^ |
|  | AGP | 37.5^a^ | 62.5 | 0^b^ | 0^b^ |  |  | AGP | 25 | 37.5^a^ | 37.5 | 0^b^ |
|  | Selacid GG | 0^c^ | 57.1 | 28.6^a^ | 14.3^a^ |  |  | Selacid GG | 14.3 | 28.6^a^ | 42.9 | 14.3^a^ |
|  | *P-value* | <0.0001 | 0.499 | <0.0001 | 0.001 |  |  | *P-value* | 0.169 | 0.02 | 0.405 | 0.001 |
| Pen – Strep (15/15µg) | Control | 75 | 25 | 0 | 0 |  | Norfloxacin (10µg) | Control | 12.5 | 50^a^ | 37.5^b^ | 0^b^ |
|  | AGP | 87.5 | 12.5 | 0 | 0 |  |  | AGP | 12.5 | 25^b^ | 62.5^a^ | 0^b^ |
|  | Selacid GG | 85.7 | 14.3 | 0 | 0 |  |  | Selacid GG | 14.3 | 28.6^b^ | 42.9^ab^ | 14.3^a^ |
|  | *P-value* | 0.576 | 0.071 |  |  |  |  | *P-value* | 0.921 | 0.005 | 0.026 | <0.0001 |
| Flo – Doxy (40/20µg) | Control | 62.5^a^ | 25^b^ | 12.5 | 0 |  | Enrofloxacin (5µg) | Control | 37.5 | 25^a^ | 25^b^ | 12.5^a^ |
|  | AGP | 25^b^ | 62.5^a^ | 12.5 | 0 |  |  | AGP | 25 | 0^b^ | 62.5^a^ | 12.5^a^ |
|  | Selacid GG | 42.9^a^ | 42.9^a^ | 14.3 | 0 |  |  | Selacid GG | 28.6 | 14.3^a^ | 57.1^a^ | 0^b^ |
|  | *P-value* | <0.0001 | <0.0001 | 0.921 |  |  |  | *P-value* | 0.256 | <0.0001 | <0.0001 | 0.002 |
| Flumequin (30µg) | Control | 50 | 25 | 25 | 0 |  |  |  |  |  |  |  |
|  | AGP | 37.5 | 37.5 | 25 | 0 |  |  |  |  |  |  |  |
|  | Selacid GG | 42.9 | 28.6 | 28.6 | 0 |  |  |  |  |  |  |  |
|  | *P-value* | 0.405 | 0.256 | 0.848 |  |  |  |  |  |  |  |  |

^a–b^ values in a column with no common superscripts differ significantly (*P* ≤ 0.05). *R: Resistant, LS: Low Susceptible, IS: Intermediate Susceptible, HS: High Susceptible*

**Appendix Table 2.** Antibiotic resistant of *E. coli* isolated in feces of finishing pigs, %

| Antibiotics | Treatment | R | LS | IS | HS | Antibiotics | Treatment | R | LS | IS | HS |
| --- | --- | --- | --- | --- | --- | --- | --- | --- | --- | --- | --- |
| Colistin (10 µg) | Control | 100 | 0 | 0 | 0 | Cefotaxime (30µg) | Control | 25^a^ | 37.5 | 25^b^ | 12.5^b^ |
|  | AGP | 100 | 0 | 0 |  |  | AGP | 25^a^ | 25 | 50^a^ | 0^c^ |
|  | Selacid GG | 100 | 0 | 0 | 0 |  | Selacid GG | 0^b^ | 42.9 | 28.6^b^ | 28.6^a^ |
|  | *P-value* |  |  |  |  |  | *P-value* | <0.0001 | 0.091 | 0.005 | <0.0001 |
| Amox – COlistin (10/10µg) | Control | 100 | 0 | 0 | 0 | Ceftiofur (30µg) | Control | 50^a^ | 25^a^ | 25^a^ | 0 |
|  | AGP | 100 | 0 | 0 | 0 |  | AGP | 62.5^a^ | 25^a^ | 12.5^b^ | 0 |
|  | Selacid GG | 100 | 0 | 0 | 0 |  | Selacid GG | 28.6^b^ | 71.4^b^ | 0^c^ | 0 |
|  | *P-value* |  |  |  |  |  | *P-value* | 0.002 | <0.0001 | <0.0001 |  |
| Amoxicillin/clavulanic acid (20/10µg) | Control | 37.5^a^ | 62.5^a^ | 0^c^ | 0 | Ciprofloxacin (5µg) | Control | 62.5 | 12.5^b^ | 12.5^a^ | 12.5^a^ |
|  | AGP | 50^a^ | 37.5^b^ | 12.5^b^ | 0 |  | AGP | 50 | 37.5^a^ | 0^c^ | 12.5^a^ |
|  | Selacid GG | 0^b^ | 71.4^a^ | 28.6^a^ | 0 |  | Selacid GG | 57.1 | 14.3^b^ | 28.6^a^ | 0^b^ |
|  | *P-value* | <0.0001 | 0.04 | <0.0001 |  |  | *P-value* | 0.499 | <0.0001 | <0.0001 | 0.002 |
| Pen – Strep (15/15µg) | Control | 87.5 | 12.5^a^ | 0 | 0 | Norfloxacin (10µg) | Control | 62.5^ab^ | 12.5^b^ | 25^a^ | 0 |
|  | AGP | 87.5 | 12.5^a^ | 0 | 0 |  | AGP | 75.0^a^ | 12.5^b^ | 0^c^ | 12.5 |
|  | Selacid GG | 100 | 0^b^ | 0 | 0 |  | Selacid GG | 42.9^b^ | 42.9^a^ | 14.3^b^ | 0 |
|  | *P-value* | 0.567 | 0.02 |  |  |  | *P-value* | 0.013 | <0.0001 | <0.0001 | <0.0001 |
| Flo – Doxy (40/20µg) | Control | 62.5^ab^ | 37.5^a^ | 0^b^ | 0 | Enrofloxacin (5µg) | Control | 50 | 25^a^ | 12.5^b^ | 12.5 |
|  | AGP | 75.0^a^ | 12.5^b^ | 12.5^a^ | 0 |  | AGP | 62.5 | 12.5^b^ | 12.5^b^ | 12.5 |
|  | Selacid GG | 42.9^b^ | 42.9^a^ | 14.3^a^ | 0 |  | Selacid GG | 57.1 | 0^c^ | 28.6^a^ | 14.3 |
|  | *P-value* | 0.013 | <0.0001 | 0.01 |  |  | *P-value* | 0.499 | <0.0001 | 0.008 | 0.921 |
| Flumequin (30µg) | Control | 75 | 12.5 | 12.5 | 0^b^ |  |  |  |  |  |  |
|  | AGP | 62.5 | 25 | 12.5 | 0^b^ |  |  |  |  |  |  |
|  | Selacid GG | 57.1 | 14.3 | 14.3 | 14.3^a^ |  |  |  |  |  |  |
|  | *P-value* | 0.273 | 0.071 | 0.921 | <0.0001 |  |  |  |  |  |  |

^a–b^ values in a column with no common superscripts differ significantly (*P* ≤ 0.05). *R: Resistant, LS: Low Susceptible, IS: Intermediate Susceptible, HS: High Susceptible*
